# Hemoptysis in a patient with MDR-tuberculosis: successful diagnosis with photon counting CT and embolization of a Rasmussen aneurysm

**DOI:** 10.1007/s15010-025-02660-3

**Published:** 2025-10-09

**Authors:** Lukas van de Sand, Benedikt M. Schaarschmidt, Johannes Wienker, Oliver Witzke, Markus Zettler

**Affiliations:** 1 Department of Infectious Diseases, University Hospital Essen, University Duisburg-Essen, Hufelandstraße 55, Essen, 45147 Germany; 2 Institute of Diagnostic and Interventional Radiology and Neuroradiology, University Hospital Essen, University Duisburg- Essen, Essen, Germany; 3 Department of Pulmonary Medicine, University Hospital Essen- Ruhrlandklinik, University Duisburg-Essen, Essen, Germany

**Keywords:** MDR-TB, Rasmussen aneurysm, Hemoptysis, Photon counting CT

## Abstract

**Background:**

Multidrug-resistant tuberculosis (MDR-TB) remains a significant clinical challenge and may be complicated by life-threatening hemoptysis. One rare but serious cause of hemoptysis in TB patients is the development of pulmonary artery pseudoaneurysms, known as Rasmussen aneurysms, which typically arise within or adjacent to tuberculous cavitary lesions.

**Case presentation:**

We report the case of a 57-year-old male patient who was diagnosed with MDR-TB in July 2024, confirmed by phenotypic resistance against rifampicin and isoniazid. According to WHO recommendations treatment with the BPaLM regimen (bedaquiline, pretomanid, linezolid, and moxifloxacin) was initiated in early August 2024 and was administered according to an extended 9-month schedule due to clinical considerations. After approximately seven months of therapy, the patient was re-hospitalized in March 2025 due to hemoptysis. A thoracic photon counting CT scan revealed regressing bilateral cavitary lesions. During the same month, a pseudoaneurysm arising from a subsegmental pulmonary artery within a cavity—consistent with a Rasmussen aneurysm—was identified. Successful embolization of the feeding vessel was performed under angiographic guidance. Post-interventional bronchoscopy showed minimal residual bloody secretions at the embolization site but no evidence of active bleeding after thorough irrigation. At that time, pending cultures for *M. tuberculosis* finally converted negative. The patient recovered well, and no further hemoptysis occurred.

**Conclusions:**

This case highlights the importance of considering Rasmussen aneurysms as a potential cause of hemoptysis in patients with cavitary MDR-TB, even several months after starting antibiotic therapy. Prompt imaging-based diagnosis and endovascular intervention are critical to avoid life-threatening complications.

## Introduction

Tuberculosis (TB) remains a major global health threat, especially in regions with limited resources. Despite advances in diagnostics and therapeutics, TB continues to claim millions of lives annually [1]. The emergence of multidrug-resistant tuberculosis (MDR-TB), defined as resistance to at least isoniazid and rifampicin - the two most potent first-line anti-TB drugs - has further complicated treatment and control efforts. MDR-TB poses significant clinical challenges, often requiring prolonged treatment with second-line agents that are less effective and associated with higher toxicity [2].

Recently, the introduction of the BPaLM regimen - comprising bedaquiline, pretomanid, linezolid, and moxifloxacin - has shown promise as a shorter, all-oral treatment option for MDR-TB [2–4]. A 24-week course of BPaLM demonstrated non-inferiority compared to conventional regimens, with an improved safety profile and fewer adverse events [4] and is currently WHO recommended treatment option for MDR-TB [5]. However, the long-term clinical outcomes and complication risks under this regimen are still under investigation.

One of the severe complications of pulmonary TB is life-threatening hemoptysis, which is typically of arterial origin and demands urgent intervention [6, 7]. Rasmussen’s aneurysm, a rare but critical vascular complication, refers to a pseudoaneurysmal dilatation of a branch of the pulmonary artery in proximity to a tuberculous cavity [8]. Its rupture can result in massive hemoptysis and high mortality if not promptly recognized and managed [6, 7].

We report a rare case of a patient with MDR-TB complicated by Rasmussen’s aneurysm—a combination that has been scarcely described in the literature. Notably, this life-threatening complication occurred approximately seven months after the initiation of appropriate MDR-TB therapy with the BPaLM regimen, highlighting the need for continued vigilance even during treatment.

## Case presentation

**Fig. 1:**
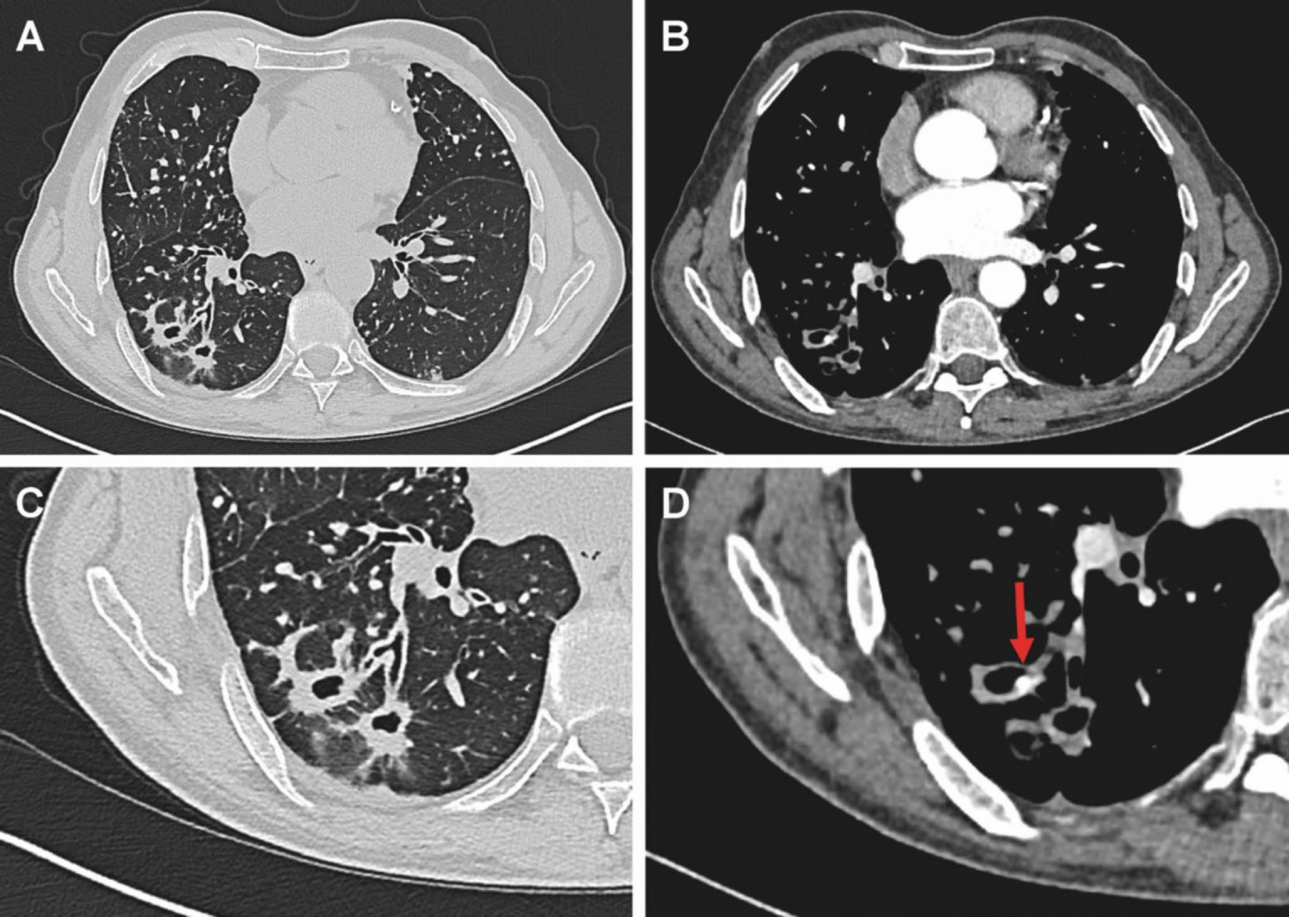
CT imaging of a patient with bipulmonary tuberculosis and cavitary pulmonary disease. Axial non-contrast (A) and contrast-enhanced CT (B) showing bilateral miliary nodules and multiple cavitary lesions in the right lower lobe with progressive consolidation of the cavities. C Magnified axial view highlighting the partially consolidated cavitary lesions in the right lower lobe. D Contrast-enhanced axial CT with detection of a pseudoaneurysm (red arrow) within a cavity in the right lower lobe, originating from a subsegmental branch of the pulmonary artery — indicating a potential source of hemoptysis.

We report the case of a 57-year-old male diagnosed with multidrug-resistant tuberculosis (MDR-TB) in July 2024, confirmed by phenotypic resistance against rifampicin and isoniazid. Tuberculostatic treatment with the BPaLM regimen (bedaquiline, pretomanid, linezolid, and moxifloxacin) was initiated in early August 2024 and was pursued as part of a prolonged 9-month regimen. After approximately seven months of ongoing therapy, the patient was readmitted in March 2025 due to hemoptysis. The patient initially presented to our infectious diseases outpatient clinic in the last week of February 2025 with the onset of hemoptysis. At this visit, the treating team decided against the originally planned discontinuation of therapy and opted instead to continue the oral regimen, with prompt scheduling for further diagnostic evaluation. He had been receiving MDR-TB treatment for nearly 30 weeks at that time, following inpatient initiation of therapy. Sputum culture converted negative two weeks after starting antibiotic therapy. Despite the hemoptysis, he described his general condition as acceptable, though he reported persistent fatigue. He denied fever, nausea, or vomiting. A chest CT scan showed no progression of disease compared to prior imaging and even demonstrated a reduction in the size of bilateral pulmonary cavities in the upper lobes and multiple cavitary lesions in the right lower lobe (Fig. 1). Symptomatic treatment with inhalation of tranexamic acid and saline was started. However, without improvement, a CT angiography (CTA) of the thorax using a photon counting CT-scanner (PCCT, Naeotom Alpha, Siemens Healthineers, Erlangen, Germany) was performed to visualize a potential bleeding from a bronchial artery (Fig. 1).

**Fig. 2:**
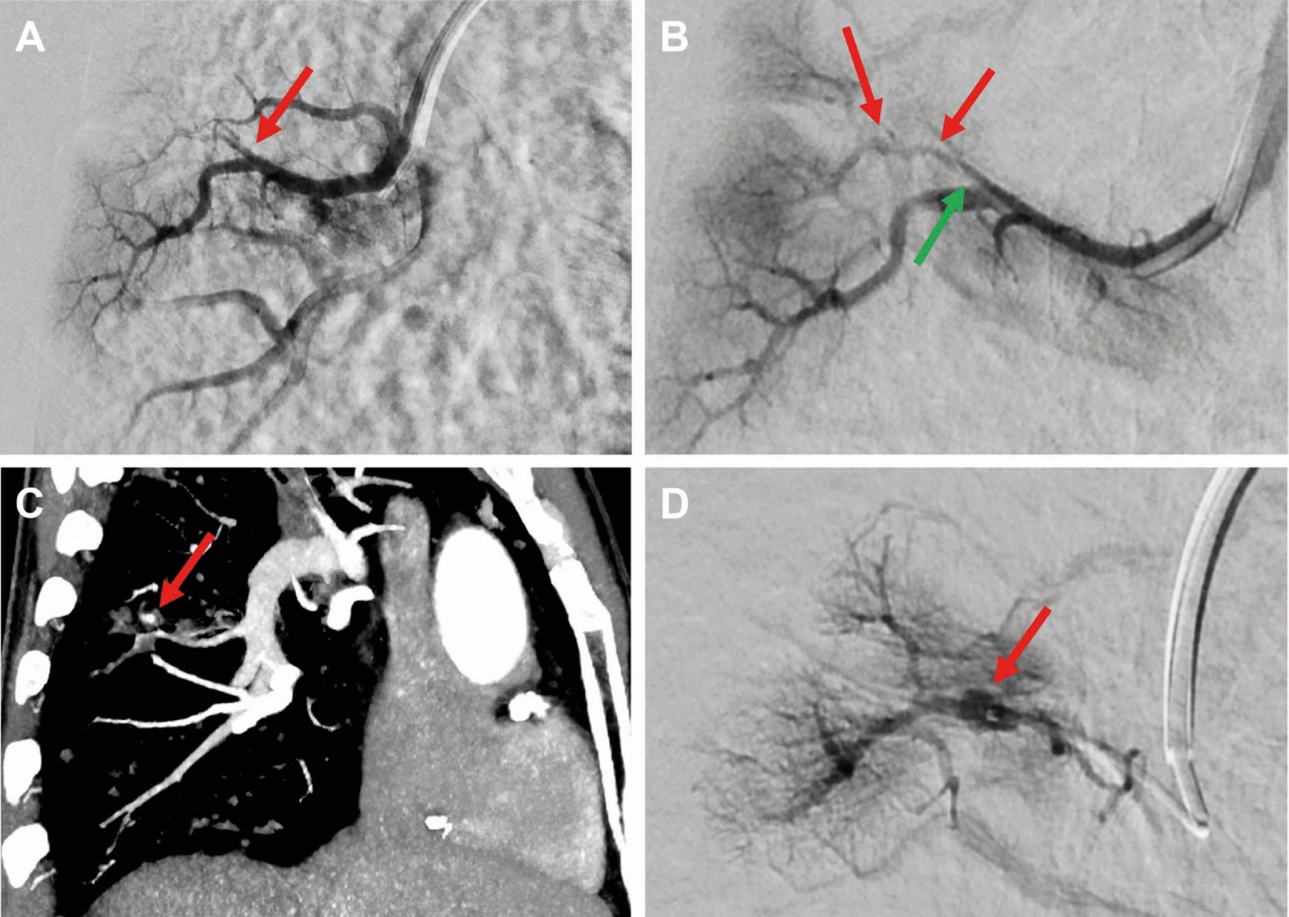
Imaging of pulmonary artery embolization in a patient with Rasmussen aneurysm. **A** Digital subtraction angiography (DSA) showing the right lower pulmonary artery and the extensive alterations of the subsegmental branch without depiction of a Rasmussen aneurysm (red arrow). **B** DSA after microcatheter placement proximal to the aneurysm bearing subsegmental branch (green arrow). Again, the aneurysm is not depicted (red arrow). **C** Contrast-enhanced CT angiography (CTA) in maximum intensity projection of the right lower pulmonary artery, here, the red arrow highlights the cavitary lesions with adjacent Rasmussen aneurysm. **D** DSA images after microcatheter placement in the subsegmental branch verify the Rasmussen Aneurysm in the suspected subsegmental branch

**Fig. 3:**
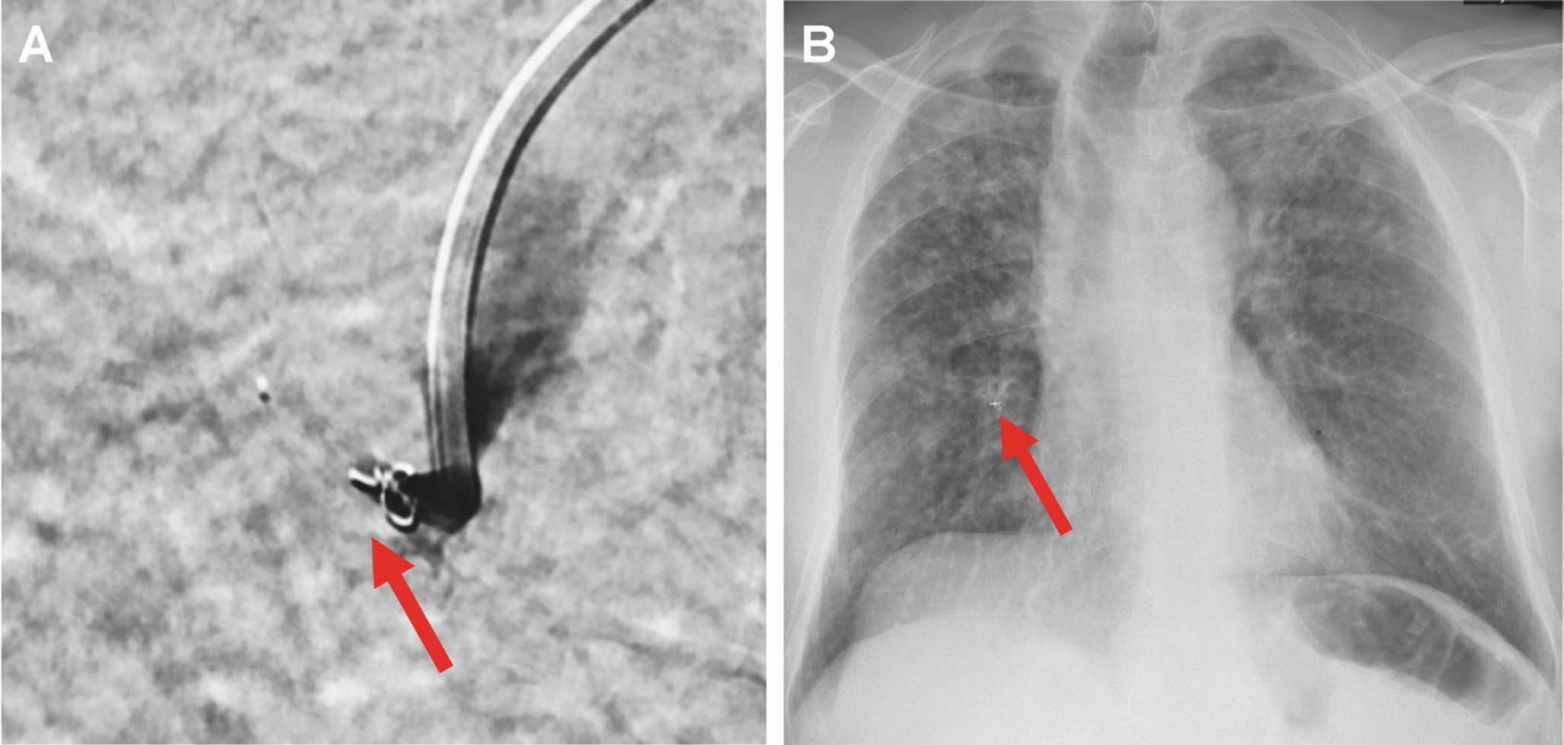
**A Fluoroscopic image** at the end of the intervention showing successful embolization of a Rasmussen aneurysm. A microvascular plug and a coil (red arrow) were deployed via a microcatheter into the targeted branch of the right pulmonary artery. Following plug and coil placement, selective contrast injection demonstrates complete occlusion of the aneurysmal vessel without residual filling — confirming the technical success of the embolization. **B Post-interventional chest X-ray** showing largely unchanged bilateral pulmonary opacities consistent with known cavities and infiltrates in the context of miliary tuberculosis. Newly visible foreign material (red arrow) is present in the right mid-lung field following embolization of a branch of the right pulmonary artery. No other significant interval changes are observed

Despite the suboptimal contrast in the pulmonary arteries, photon counting CT revealed a pseudoaneurysm within a cavity in the right lower lobe, arising from a subsegmental branch of the pulmonary artery. This is a typical presentation of a Rasmussen aneurysm. After interdisciplinary consultation, this vascular lesion was identified as the most probable bleeding source and the decision for interventional embolization was made. Vascular access was obtained via the right common femoral vein. Under fluoroscopic guidance, a 7 F sheath (Super Arrow-Flex, Teleflex, Wayne, PA, USA) and a 7 F guiding catheter (Guider Softtip XF, Kalamazoo, MI, USA) were advanced via a J-tip guide wire to the right atrium. Then, access to the pulmonary trunk was obtained using a 155° angled pigtail catheter. Then, a 4 F vertebral catheter was advanced to the right lower pulmonary artery using a steerable SplashWire (Merit Medical Systems, Inc., South Jordan, UT, USA). Here, contrast-enhanced images were acquired, but no aneurysm could be visualized as multifocal stenoses, most probably caused by inflammatory changes, led to an incomplete contrast filling of the target vessel. Using multiplanar reformations of the PCCT images, the vascular branch was visualized and the correct catheter position was verified. Then, a microcatheter (Rebar-27, Medtronic, Dublin, Ireland) was navigated selectively into the supposedly affected pulmonary artery branch. Here, the pseudoaneurysm could be visualized (Fig. 2). The vessel supplying the pseudoaneurysm was occluded using a 5 mm microplug (MVP Micro Vascular Plug System, Medtronic, Dublin, Ireland) and a 4 mm complex helical-18 push coil (Boston Scientific, Marlborough, MA, USA). The technical success of the procedure was demonstrated by post-deployment angiography with selective contrast injection, which verified total occlusion of the aneurysmal vessel with no remaining contrast filling (Fig. 3).

Meanwhile, sputum samples were collected under isolation precautions to exclude treatment failure while on BPaLM regimen. The third sample showed acid-fast bacilli on auramine staining, while PCR confirmed *Mycobacterium tuberculosis* with rifampicin resistance, consistent with prior findings. The patient’s sputum cultures had converted to negative within two weeks of therapy, and all subsequent samples remained negative except for a single sample that tested positive on PCR testing. Pending culture results, the tuberculostatic therapy was continued without modification.

**Fig. 4:**
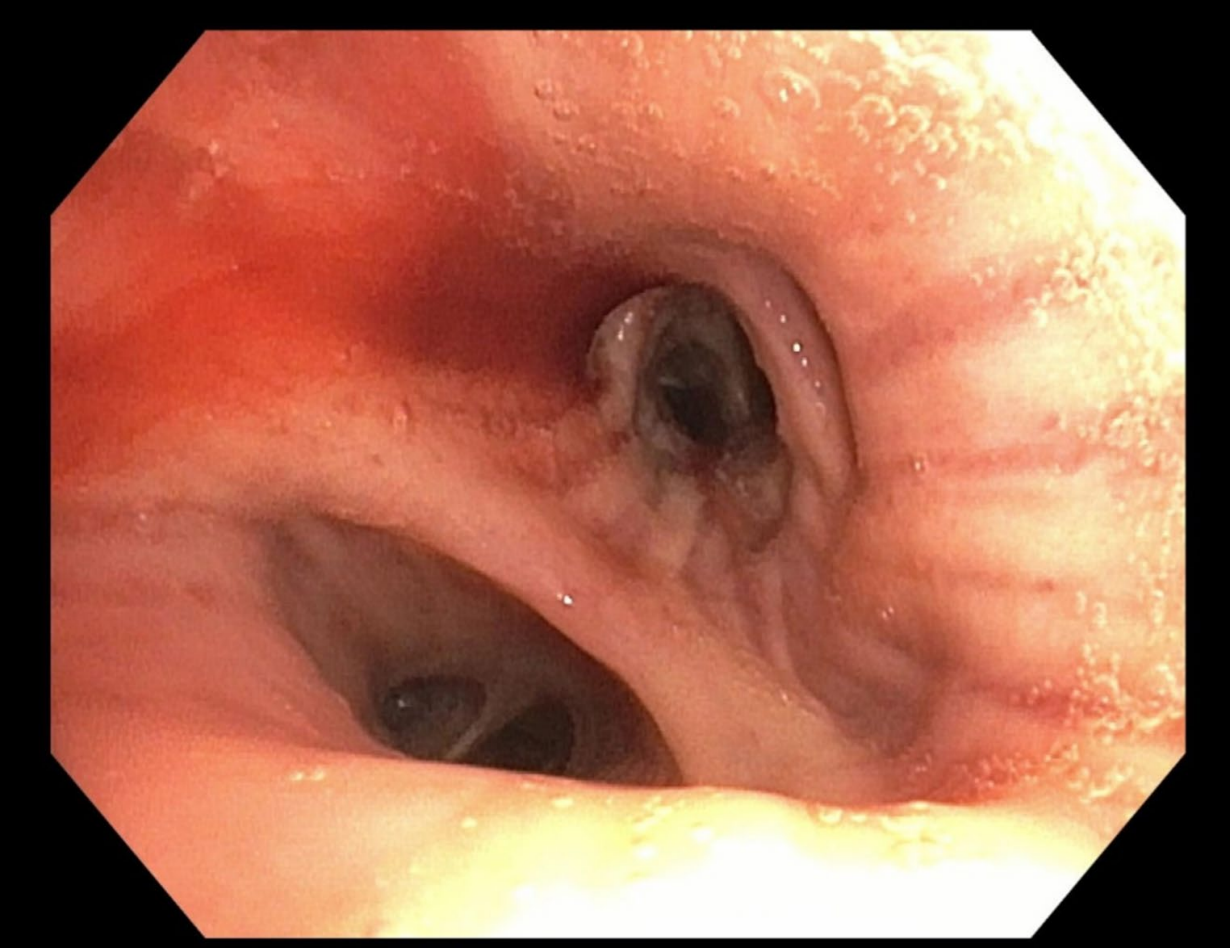
Bronchoscopy image. At the bifurcation of RB9/10 in the right lower lobe, a small amount of bloody secretion is observed at the origin of an accessory bronchial orifice. Despite thorough rinsing, there were no signs of active bleeding. The site corresponds to the location of a recently performed angiographic aneurysm occlusion. Bronchoscopic findings also showed signs of chronic bronchitis. Due to the minimal amount of secretion, no further intervention was required

At the day after embolization, the patient once again produced slightly blood-tinged sputum. In response, we performed a diagnostic bronchoscopy after about one week to pinpoint the bleeding source and obtain further microbiological samples. The bronchoscopy, performed by a specialized pulmonary team, was uneventful. Minimal bloody secretion was detected at an accessory bronchial orifice in the right lower lobe (RB 9/10), matching the location of the previously embolized pseudoaneurysm (Fig. 4). Even after intensive irrigation, no active bleeding was observed. Bronchoalveolar lavage (BAL) was performed for microbiological evaluation. Given the minimal secretion and the absence of active bleeding, there was no indication for further intervention. Microscopy of the lavage fluid showed no acid-fast bacilli, and the culture remained pending at discharge. As part of the differential diagnostic workup, we evaluated for chronic pulmonary aspergillosis (CPA). Aspergillus antigen testing via BAL was negative, and serological testing for Aspergillus-specific IgG also yielded negative results.

During the remaining inpatient period of about two weeks, no further episodes of hemoptysis were observed. The patient was discharged in the second half of March 2025 in improved general condition for continued outpatient care. He subsequently attended follow-up visits at intervals of four and eight weeks after discharge - in April and May 2025 - at our outpatient clinic. He reported stable general health, regular intake of his MDR-TB medication without significant side effects and successfully completed the 9-month course in early May 2025. Clinically, he remained free of productive cough, and no further hemoptysis occurred. Laboratory findings, including renal and hepatic function and inflammatory markers, were within normal limits. The QTc interval on ECG remained normal. Another notable comorbidity was a known ascending aortic aneurysm, that primary had a diameter of 43 mm. Throughout tuberculostatic treatment, the aneurysm was routinely observed by transthoracic echocardiography. Imaging continuously showed constant dimensions over time, with no signs of progression nor clinical symptoms related to the pseudoaneurysm.

Chest X-rays at follow-up showed stable bilateral infiltrates and cavities consistent with known bipulmonary tuberculosis (Fig. 3). Although a sputum sample obtained during the March hospitalization tested microscopically positive for acid-fast bacilli, the final culture result, available after roughly nine weeks, was negative at the end of May 2025. With this negative culture result, tuberculostatic therapy was discontinued, and the MDR-TB treatment was considered successfully completed.

## Discussion

This case presents a rare combination of MDR-TB complicated by a Rasmussen aneurysm - a constellation that is scarcely described in the literature [9]. While hemoptysis in pulmonary tuberculosis most commonly results from bronchiectasis or erosion of hypertrophied bronchial arteries, bleeding from a pulmonary artery source, known as a Rasmussen aneurysm, remains an infrequent but serious complication. Originally described by Fritz Waldemar Rasmussen in 1868, Rasmussen aneurysms are identified in only about 0.25% of patients with pulmonary tuberculosis and hemoptysis [10].

For the diagnosis of Rasmussen aneurysm, CT pulmonary angiography (CTPA) remains the gold standard, allowing precise localization of the bleeding source and vascular abnormalities [9]. However, due to the rarity of this condition, CTA is preferably performed in TBC patients to exclude bronchioarterial causes for a bleeding. In this case, the excellent image quality of PCCT allowed for the detection of this rare pulmonoarterial condition in a pulmonary CTA despite the suboptimal contrast of the pulmonary arteries [11].

Similar cases have been reported, describing life-threatening hemoptysis as a severe complication that often necessitates intubation and intensive care admission [12]. However, despite experiencing daily small amounts of hemoptysis until the embolization procedure, our patient remained clinically stable at all times. Vital parameters such as oxygen saturation and hemoglobin levels showed no critical deviations, highlighting an unusual clinical course compared to the high mortality rates of nearly 50% associated with massive hemoptysis in tuberculosis patients [13].

Several endovascular embolization methods have been tested, such as glue application, coil embolization, and stent graft placement. None of these strategies, however, have proven to be significantly superior to the others [14]. In our case, we decided to occlude the whole subsegmental branch with a microplug and an additional coil due to extensive inflammatory changes of the pseudoaneurysm bearing vessel.

Post-procedure, the patient experienced a brief episode of mild hemoptysis, a known phenomenon likely due to residual blood in the airways rather than active rebleeding. Flexible bronchoscopy is known to localize the bleeding source in up to 93% of cases [15]. In our case, bronchoscopy confirmed the anatomical correspondence between the previously embolized segment and residual blood, with no signs of active hemorrhage after extensive lavage. This finding supports the assumption that the Rasmussen aneurysm indeed represented the primary bleeding source. Both imaging and endoscopic findings in our case confirmed the effectiveness of the embolization.

Another important differential diagnosis in TB patients with hemoptysis is CPA, particularly when cavitary lesions persist. CPA can develop as a sequel to tuberculosis, with a global five-year prevalence of approximately 18 per 100,000 population [16]. Aspergilloma formation within residual cavities is a known cause of delayed hemoptysis [17]. In our case, CPA was considered due to the timing of symptoms and presence of cavitary disease. We performed BAL for Aspergillus antigen (negative) and assessed Aspergillus-specific IgG, which was also negative, effectively ruling out CPA [18]. Especially the confirmation of a pseudoaneurym on CT scan made an aspergilloma highly unlikely.

Concerns have been raised by recent epidemiological studies regarding an association between fluoroquinolone use and a higher incidence of aortic dissections and aneurysms [19–21]. While the absolute risk appears to be low in the general population, patients with pre-existing aortic pathologies may be more susceptible [22]. During echocardiographic monitoring, treatment with moxifloxacin was continued without any enlargement of the previously known aortic aneurysm being detected. Recent recommendations point out that despite the reported association between fluoroquinolones and aortic aneurysms or dissections, their use should not be categorically avoided when clinically indicated [22, 23]. However, in our patient, it cannot be conclusively ruled out that the development of the Rasmussen aneurysm may have been promoted or unmasked by moxifloxacin therapy. It remains possible that preexisting inflammatory damage from pulmonary tuberculosis, in combination with the matrix-degrading effects of fluoroquinolones, contributed to the aneurysmal transformation of a pulmonary artery branch [24].

Unlike other reported cases where patients often deteriorated despite intervention, our patient showed a stable course and responded well to therapy without the need for intensive care admission.

## Conclusion

This case emphasizes the importance of regular follow-up and close clinical monitoring of tuberculosis patients, particularly those receiving long-term treatment for MDR-TB. Complications such as Rasmussen aneurysm may arise even after months of adequate antimicrobial therapy, underlining the need for vigilance throughout the entire treatment period.

**Table:** Antibiogram

	M. tuberculosis complex
Amikacin	S
Levofloxacin	S
Moxifloxacin	S
Linezolid	S
Rifampicin	R
Isoniazid	R
Delamanid	S
Ethambutol	S
Cycloserin	S
Pretomanid	S
Protionamid	S
Bedaquilin	S

## Data Availability

The original contributions presented in the study are included in the article. Further inquiries can be directed to the corresponding author.
